# Accuracy, Quality and Readability of Knee Osteoarthritis Information on Social Media (Instagram e TikTok) and Search Platforms (Google e YouTube): A Cross‐Sectional Study

**DOI:** 10.1002/msc.70267

**Published:** 2026-10-28

**Authors:** Rayane Quintão Castro, Giovanna Ferreira de Oliveira, Marcella Nogueira Marques, Victória Beatriz Silva, Diogo Simões Fonseca, Carla Malaguti

**Affiliations:** ^1^ Programa de pós‐graduação em Saúde Faculdade de Medicina Universidade Federal de Juiz de Fora Juiz de Fora Brazil; ^2^ Programa de pós‐graduação em Ciências da Reabilitação e Desempenho Físico Funcional Faculdade de Fisioterapia Universidade Federal de Juiz de Fora Juiz de Fora Brazil; ^3^ Faculdade de Fisioterapia Universidade Federal de Juiz de Fora Juiz de Fora Brazil

**Keywords:** accuracy, knee osteoarthritis, quality, readability

## Abstract

**Background:**

Knee osteoarthritis (KOA) is a chronic condition with a high global prevalence, often resulting in functional decline. Faced with this, many people turn to the internet to find information about the disease and available treatments. Besides the amount of content available online, it does not ensure its accuracy, quality and readability. This study aims to evaluate the accuracy, quality and readability of KOA‐related information available on Instagram, TikTok, Google and YouTube in Brazilian Portuguese.

**Methods:**

Google and YouTube searches were performed using ‘Tratamento de osteoartrite de joelho’, ‘Tratamento de artrose no joelho’ and ‘Tratamento de artrite no joelho’. For each term, the first 20 results were selected. Searches on Instagram and TikTok were conducted using the hashtags: #artrosenojoelho, #artrosedojoelho, and #artrosedejoelho, selecting the first 100 posts for each term. All the research was done on June 19, 2024. Content accuracy was assessed against a reference resource on knee osteoarthritis, information quality was assessed using DISCERN, and the readability of Google content was evaluated using the Flesch–Kincaid Grade Level scale.

**Results:**

We found that the information about medical management was the most classified as inaccurate. The overall DISCERN score indicated low quality (2.3 ± 0.7) and 78% of Google texts required at least 14 years of schooling to read.

**Conclusion:**

As digital platforms have become a widely used source of health information, our findings raise concerns about the potential for misinformation to delay effective treatment and perpetuate low‐value care.

## 1 Introduction

Knee osteoarthritis (KOA) is a prevalent chronic condition that significantly affects the physical and psychosocial well‐being of individuals worldwide (Zhang et al. 2023). Due to its multifactorial and subjective nature, KOA is associated with diverse biomechanical alterations and symptoms that often result in functional impairments, making daily activities challenging and potentially unsafe (Boekesteijn et al. 2022). Faced with these difficulties, many individuals seek health‐related information online to better understand their condition and explore available treatments options (Hong et al. 2023).

Digital platforms have become a widely used source of health information, and Bibliometric and Altmetric analyses have highlighted how online engagement can complement traditional citation‐based measures of academic visibility (Özduran And Hanci 2023). Approximately 80% of internet users search for health‐related topics online, reflecting a growing tendency towards self‐directed health management (Ferreira et al. 2019; Forgie et al. 2021). This role became particularly evident during the COVID‐19 pandemic, when many professionals and non‐professionals turned to the Internet to obtain information about health conditions, including diagnosis and treatment (Özduran and Hancı 2022). Youtube, for example, is widely used as a source of information, having been accessed by 73% of adults in the United States (Şan 2021). The convenience and accessibility of these platforms often exceed traditional clinical channels, promoting a participatory environment where users not only consume information but also engage in community‐based support networks (Ferreira et al. 2019; Hong et al. 2023). These interactions can enhance trust in and adherence to shared health information.

Despite the popularity of online sources, the abundance of available content does not guarantee its accuracy, or reliability. Research examining KOA‐related websites has revealed that much of the content is of low‐quality, with significant inconsistencies in scope, credibility, and readability (Goff et al. 2023). In particular, content frequently lacks clarity and alignment with established clinical guidelines, especially concerning first‐line treatments, not only in concern about KOA, but also about thoracic outlet syndrome (Özbek 2025), low back pain (Ferreira et al. 2019), failed back spinal surgery (Guo et al. 2019), gout and urate‐lowering (Jimenez‐Liñan et al. 2017). As many users cannot critically evaluate the reliability of online information, exposure to inaccurate or misleading content may negatively influence treatment decisions and health outcomes(Hong et al. 2023; Özbek et al. 2025).

Given the increasing role of digital media in health communication, it is crucial to assess the accuracy, quality and readability of KOA‐related information accessible via social media platforms and search engines. Instagram, TikTok, Google and YouTube were selected because they are widely used sources of health information and represent different formats of online content, including text and short‐ and long‐form videos. This study therefore aimed to evaluate KOA‐related content across these platforms, contributing to efforts to promote evidence‐based health education and minimise the risks associated with misinformation.

## 2 Methods

### 2.1 Study Design

This study used a cross‐sectional observational design to assess the accuracy, quality, and readability of information about knee osteoarthritis available on social media and search platforms.

### 2.2 Search Strategy

Searches were conducted across different platforms (Google, YouTube, Instagram and TikTok) on June 19, 2024. For Google and YouTube, searches were performed using an anonymous browser window, without signing into Google or YouTube accounts, to minimise algorithmic personalisation. The following Portuguese‐language keywords were used: ‘Tratamento de osteoartrite de joelho’, ‘Tratamento de artrose no joelho’ and ‘Tratamento de artrite no joelho’. On Instagram and TikTok, the search was conducted using the hashtags: #artrosenojoelho, #artrosedojoelho, and #artrosedejoelho.

The number of results selected per platform was defined a priori using a pragmatic sampling approach designed to reflect the content most readily accessible to a typical user during routine searches. For Google and YouTube, we analysed the first 20 results for each search term because users tend to concentrate their attention on the highest‐ranked results. For Instagram and TikTok, we selected the first 100 posts for each hashtag to obtain a broader sample of short‐form social media content while maintaining the feasibility of manual assessment. This sampling strategy was intended to provide a cross‐sectional overview of highly visible KOA‐related content rather than an exhaustive representation of all available posts or pages.

### 2.3 Data Extraction and Coding

Content accuracy was evaluated using the framework proposed by Goff et al. (2022), which outlines evidence‐based recommendations related to knee anatomy, pathophysiology, risk factors, epidemiology, diagnosis, symptoms, the biopsychosocial model of pain, self‐management strategies, physical activity, therapeutic exercise, weight management, surgical interventions, pharmacological treatments, and adjunct therapies.

Each post/video was evaluated individually. For every domain addressed within a given post, a single overall classification was assigned (accurate and clear, partially accurate, inaccurate, or not mentioned), according to the predefined criteria. Thus, classification was performed at the post level within each domain. The ‘not mentioned’ category was used when a post referred to a given domain (e.g., diagnosis) but did not present the specific evidence‐based criteria outlined in the assessment framework.

Two independent evaluators conducted the assessments. Both were licenced physiotherapists with clinical experience in musculoskeletal conditions, including knee osteoarthritis. One evaluator is currently enroled in a Master's programme, and the other is currently pursuing a PhD. In cases of disagreement, a third evaluator reviewed the content and provided the final judgement.

The study was designed as a descriptive cross‐sectional analysis. Therefore, no inferential statistical comparisons were performed between accuracy categories across platforms or content domains.

### 2.4 Information Quality

Information quality was assessed using the DISCERN instrument developed by Charnock et al. (1999), which includes 16 standardized items focused on the quality of written information regarding treatment choices. Each item is rated on a five‐point Likert scale ranging from ‘No’ (1) to ‘Yes’ (5), reflecting the degree to which each criterion is met. The DISCERN tool evaluates not only the accuracy or comprehensiveness of the content but also the clarity with which the benefits and risks of each treatment option are presented. These elements are critical for individuals with knee osteoarthritis to make informed decisions about their treatment options (Charnock et al. 1999). For video‐based content, DISCERN items were evaluated based on the spoken information presented in the video, as well as any accompanying textual information (e.g., video descriptions, captions, or subtitles) when available.

The readability of the textual content retrieved from Google was assessed using the online tool Legibilidade (https://legibilidade.com/), which applies multiple readability indices adapted for the Portuguese language. The following indices were analysed: Flesch Reading Ease, Flesch–Kincaid Grade Level, Gulpease Index, Gunning Fog Index (adapted), Automated Readability Index (ARI), and Coleman–Liau Index. These indices estimate readability based on linguistic features such as average sentence length, word length, syllable count, and proportion of complex words. While some indices (e.g., Flesch Reading Ease and Gulpease Index) provide a score indicating ease of reading, others (e.g., Flesch–Kincaid Grade Level, Automated Readability Index, Coleman–Liau, and Gunning Fog Index) estimate the educational level required to understand the text. For interpretation in relation to the sixth‐grade reading level, a Flesch Reading Ease Score (FRES) of 80 or higher and scores of 6 or lower for grade‐level formulas are considered consistent with the recommended readability level. Thus, lower FRES scores and higher scores on grade‐level formulas indicate greater reading difficulty (Kara et al. 2025). The software applies language‐specific adaptations for Portuguese, including adjustments in syllable calculation and definition of complex words, which are typically defined as words with three or more syllables. All texts were extracted in full and analysed without modification, except for the removal of non‐textual elements (e.g., emojis, hyperlinks, and formatting artefacts) when necessary (Moreno et al. 2023). Readability assessment was limited to Google‐derived content, as established readability formulas are validated for continuous written text and are not methodologically appropriate for predominantly audiovisual or short‐form social media content.

## 3 Results

Of the 60 links retrieved from Google, 22 were duplicated, unrelated to knee osteoarthritis, or no longer available. On YouTube, of the 60 links identified, 33 were duplicated or were excluded for not being relevant to the topic. The TikTok searches produced 127 duplicated posts or unrelated to the scope of this study. Finally, among the 300 Instagram posts, 43 were duplicated or discussed about different conditions or body regions. The final number of analysed contents was: Google (*n* = 38), YouTube (*n* = 27), TikTok (*n* = 173), and Instagram (*n* = 257).

### 3.1 Accuracy

A platform‐specific summary of information accuracy, categorised as accurate and clear, partially accurate, inaccurate, or not mentioned, is presented in Figure 1.

**FIGURE 1:**
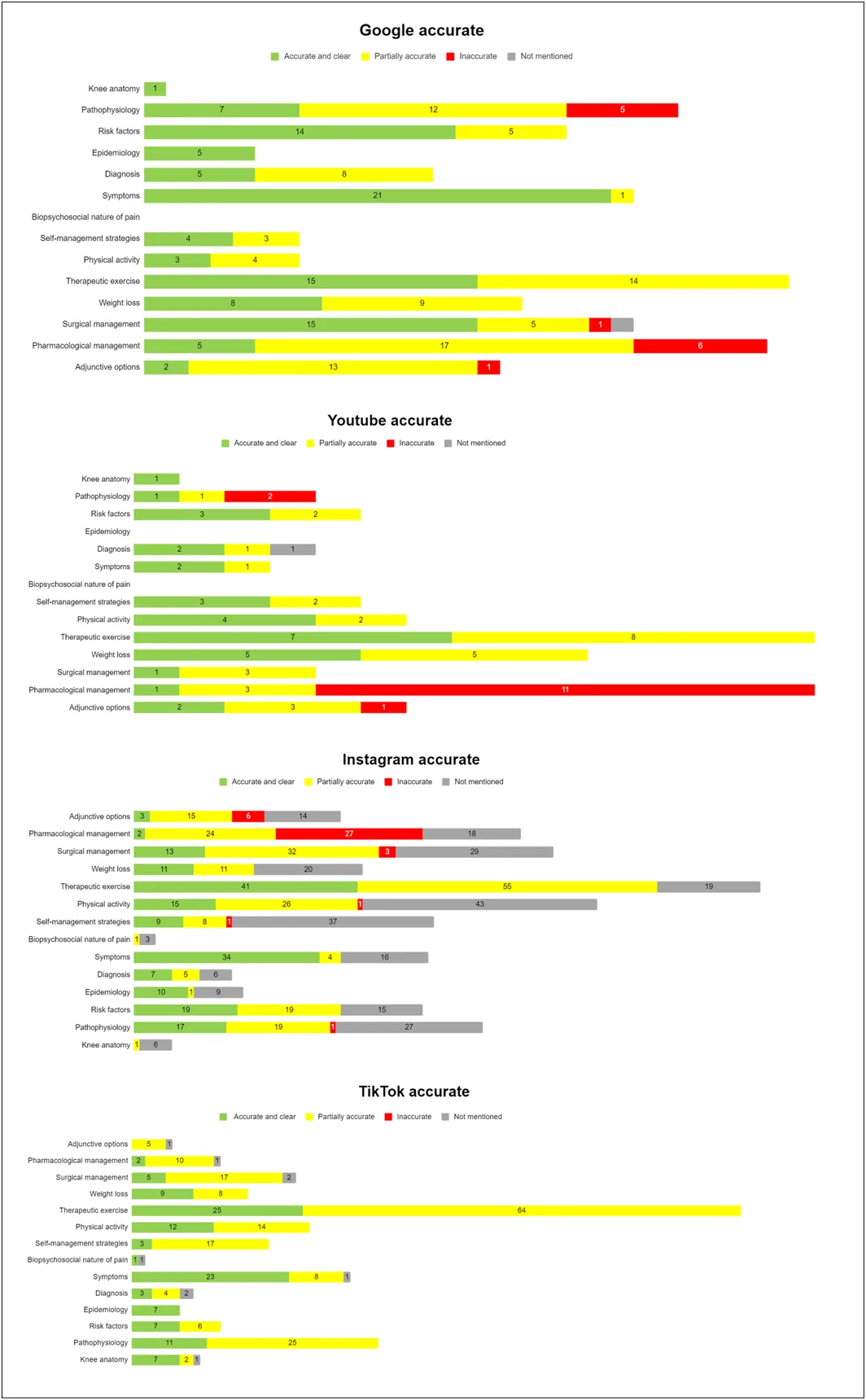
Distribution of accuracy classifications per post within each domain across Google, YouTube, Instagram, and TikTok. Each post was assigned a single classification per domain.

### 3.2 Quality

Using the DISCERN tool, the mean overall quality scores were 3.32 for Google, 2.80 for YouTube, 2.20 for Instagram, and 2.15 for TikTok. Figure 2 details the mean score for each DISCERN item across the four platforms, highlighting specific strengths (e.g., clarity of treatment aims) and weaknesses (e.g., balanced presentation of benefits and risks).

FIGURE 2Mean DISCERN item scores for Google, YouTube, Instagram, and TikTok.

**Figure:**
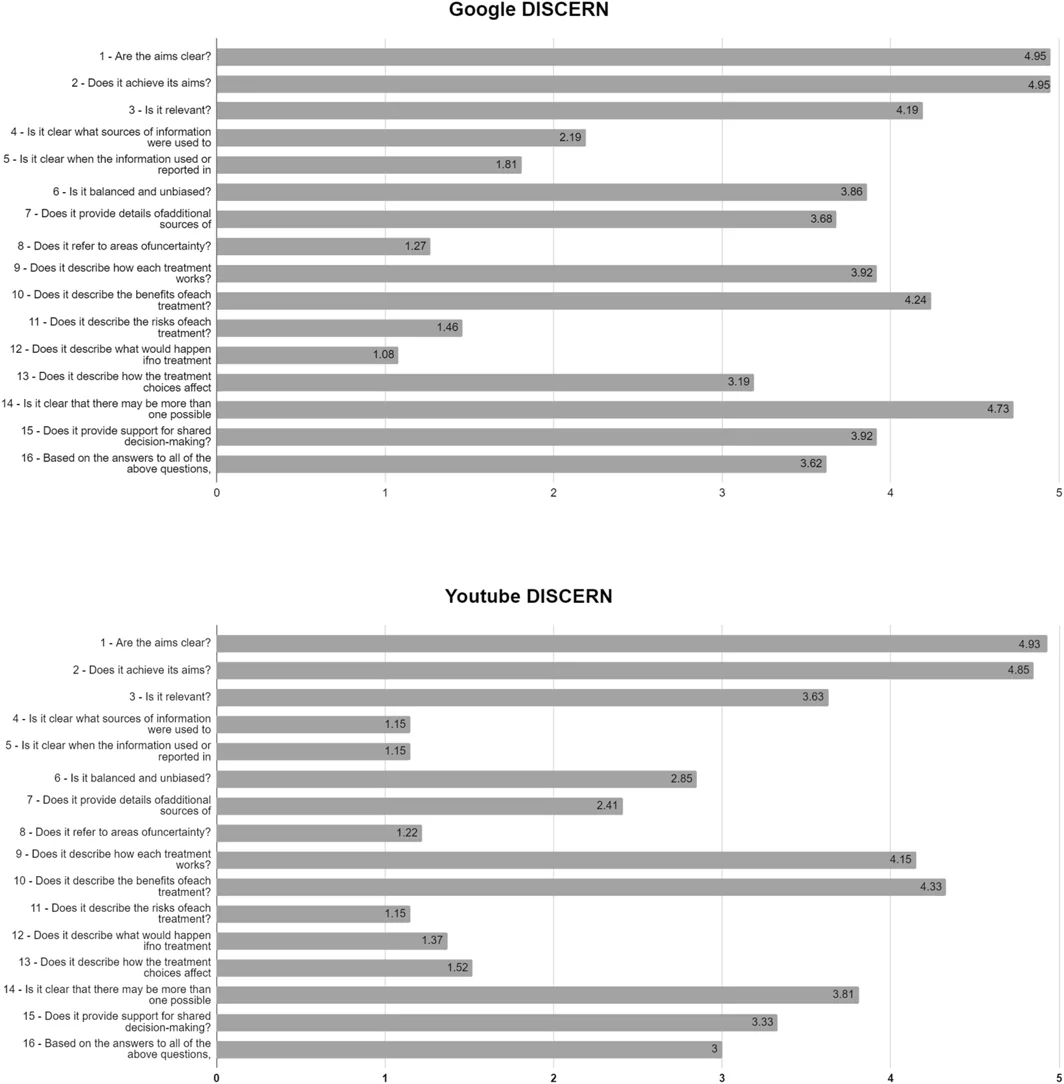

**Figure:**
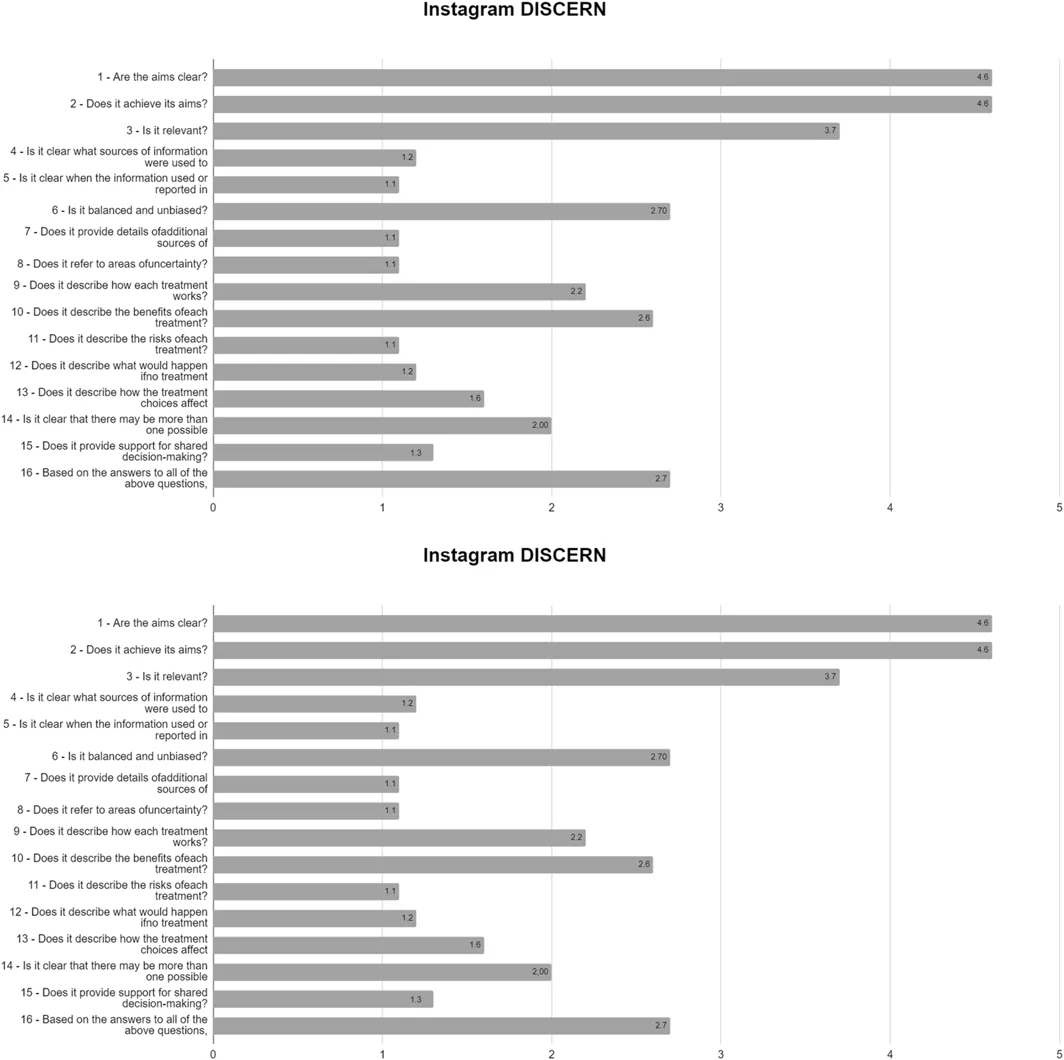

### 3.3 Readability

A total of 29.7% of the content reached a readability level of 15, classified as moderately difficult, and generally understandable by readers with a university‐level education. The full distribution of readability levels is summarised in Table 1.

**TABLE 1:** Readability distribution of google content.

Readability level	*N* (%)
Level 11. High readability. Simple text. Suitable for teenagers aged 16 to 17.	2 (5.4)
Level 12. High readability. Simple text. Suitable for teenagers aged 17 to 18.	6 (16.2)
Level 13. Moderate readability. Medium difficulty. Can be well understood by early‐year university students.	3 (8.1)
Level 14. Moderate readability. Medium difficulty. Can be well understood by university students.	9 (24.3)
Level 15. Moderate readability. Medium difficulty. Can be well understood by university students.	11 (29.7)
Level 16. Moderate readability. Medium difficulty. Can be well understood by final‐year university students.	2 (5.4)
Level 17. Low readability. Difficult text. Suitable for individuals with higher education.	2 (5.4)
Level 18. Low readability. Difficult text. Suitable for individuals with higher education.	2 (5.4)

The description of the readability components of the content retrieved from Google searches is summarised in Table 2.

**TABLE 2:** Readability of content retrieved from google searches.

Readability components	Average (mean)	Standard deviation (SD)	Minimum value	Maximum value
Flesch reading ease test	30.5	9.4	3.4	48.5
Gulpease index	50.2	4.3	40.6	60.4
Flesch‐kincaid grading level	14.3	1.9	10.6	18.9
Adapted gunning cloudiness index	14.7	1.8	11.3	18.1
Automated readability index (ARI)	13.7	2.1	9.5	18.4
Coleman‐liau index	14.4	1.4	12.0	18.4
Number of letters	5832	3391	988	16,849
Number of syllables	2595	1499	446	7409
Number of words	1068	614	186	3070
Number of sentences	56	30	11	129
Letters/words	5.5	0.2	5.1	6.2
Syllables/words	2.4	0.1	2.3	2.8
Words/sentences	19.5	3.2	12.3	25.4
Complex words	295	193.4	51	968

## 4 Discussion

This study evaluated the accuracy, quality, and readability of knee osteoarthritis information on social media platforms (Instagram, TikTok) and search engines (Google, YouTube). We found that the information about medical management was the most classified as inaccurate, while the overall DISCERN score indicated low quality (mean = 2.3 ± 0.7) and Google texts required ≥ 14 years of schooling to read. These findings matter because people increasingly rely on the internet for health information. In a systematic review, Chou et al. (2018) showed that patients with OA consult the web whenever clinicians or printed materials unmet their information needs (Chou et al. 2018). This highlights the importance of reliable internet information about knee osteoarthritis symptoms, options of treatment and other important information.

The overall DISCERN score indicated low quality and a clear gradient across platforms—Google (3.3) out‐performed YouTube (2.8), while Instagram (2.2) and TikTok (2.1) clustered at the bottom. An observational review of the 100 most‐viewed TikTok videos on knee osteoarthritis found that fewer than one‐quarter contained evidence‐based advice and that most were produced by non‐clinicians (Hong et al. 2023). Likewise, a recent Google‐based survey of English‐language websites reported that only 35% of pages clearly described treatment options, with a median modified‐DISCERN score of 40/80—well below the ‘good’ threshold (Sullivan et al. 2022). By documenting the same deficits in Brazilian Portuguese content across both search engines and high‐engagement social platforms, our study underscores that misinformation about knee osteoarthritis is systemic, transcending language, country and media format.

Several structural factors help explain why misinformation succeeds. First, platform algorithms reward engagement rather than accuracy, amplifying commercially appealing messages about hyaluronic acid injections and nutraceuticals such as glucosamine, chondroitin, fish oil/omega‐3, and vitamin D that lack guidelines support (Conley et al. 2023; Gibbs et al. 2023). Second, the outdated ‘wear‐and‐tear’ narrative remains dominant; in our sample, nearly two‐thirds of pathophysiology posts framed KOA as progressive cartilage erosion, which discourages exercise because of fears of further joint damage (Morgan et al. 2024). This matches with our search for physical‐activity content, where 187 posts were only partially accurate versus 122 accurate. This may model patient attitudes and a qualitative work has documented a general scepticism towards first‐line conservative care when information is unclear or contradictory (Battista et al. 2022). Third, comprehension barriers increase the problem: 78% of Google texts required at least 14 years of schooling according to grade‐level readability indices (e.g., Flesch–Kincaid Grade Level, Gunning Fog, Automated Readability Index, and Coleman–Liau), whereas the recommendation is that health materials not exceed a sixth‐to eighth‐grade reading level (Rooney et al. 2021). Given that Brazilians aged 60 or older had an average of 5.0 years of formal education, many users are effectively excluded from trustworthy sources and left with inaccurate persuasive alternatives, perpetuating low‐value care and delayed uptake of evidence‐based interventions (IBGE 2016). Given that online health information is accessed by individuals with different educational and socioeconomic backgrounds, presenting information in clear and simple language is essential to support understanding of disease processes and treatment options (Erkin et al. 2026). Moreover, our findings are consistent with evidence from other languages. Özduran et al. found that Turkish internet‐based patient education materials on low back pain had low reliability and poor quality. These findings suggest that difficulties in the readability and quality of online health information are not limited to English‐language content (Ozduran et al. 2024).

Our findings carry actionable implications for KOA care. Clinicians should routinely ask where patients obtain KOA information and provide a concise, clinician‐reviewed list of Portuguese resources. Professional societies and universities can scale this effort by co‐creating short videos and infographics written at—or below—the sixth‐to eighth‐grade reading level recommended for patient materials (Badarudeen and Sabharwal 2010). Complementing these measures with digital‐literacy workshops in primary‐care visits would further limit exposure to low‐value treatments and promote earlier adoption of guideline‐recommended exercise, education, and weight‐management strategies. More recently, AI‐based tools have emerged as additional sources of health information and exercise guidance for people with chronic conditions. However, the quality and reliability of such information may vary (Özbek and Özduran 2025).

This study has limitations that warrant caution in interpreting the findings. First, the number of results selected for each platform was based on a pragmatic sampling strategy intended to capture the most visible and readily accessible content, rather than all available KOA‐related information. Although this approach reflects typical user exposure, it may have limited the representativeness of the sample. Second, the data represent a single snapshot captured on 19 June 2024; because online content changes dynamically, recency may have influenced both the quality and accuracy of the information retrieved. Older content may remain highly visible despite being outdated, while newer evidence‐based content may have been underrepresented. Third, although we used incognito mode for Google and YouTube, platform algorithms may have still been influenced by location, device metadata, and other non‐controlled factors. Fourth, we did not stratify analyses according to content creator characteristics (e.g., health professional vs. non‐health professional), engagement metrics (e.g., views, likes, shares, comments), or funding/commercial interests, all of which may influence both the dissemination and credibility of content. Fifth, we did not perform inferential statistical comparisons between accuracy categories across platforms or domains; thus, our findings should be interpreted as descriptive rather than comparative. In addition, readability was assessed using a single online calculator, which may introduce measurement variability compared with other readability calculators. Finally, DISCERN and readability tools have inherent limitations when applied to audiovisual and social media content.

A strength of this study is the evaluation of KOA‐related information across multiple widely used digital platforms, including social media and search platforms. In addition, the assessment of accuracy, quality, and readability provided a multidimensional evaluation of the information available to users. Future work should include longitudinal monitoring to track how KOA content evolves as platforms update their algorithms and as clinicians respond with evidence‐based posts. Second, interventional studies are needed to test pragmatic solutions, such as clinician‐endorsed ‘KOA‐Info Seal’ labels, sixth‐grade–level infographics co‐designed with patients, or in‐app prompts that direct users to guideline‐aligned exercise videos—and to measure their impact on knowledge, treatment choice and clinical outcomes. Third, studies should also stratify analyses by author credentials, content format, funding source and algorithmic ranking signals to clarify how each factor influences trust and reach. By coupling continuous monitoring with user‐centred interventions and supportive policy frameworks, the healthcare community can turn today's fragmented digital landscape into a credible, accessible and empowering ecosystem for people living with knee osteoarthritis.

## 5 Conclusion

Our study reveals that online information about knee osteoarthritis published in Brazilian Portuguese on social media platforms, is often inaccurate, of low quality, and difficult to read. As digital platforms have become a widely used source of health information, our findings raise concerns about the potential for misinformation to delay effective treatment and perpetuate low‐value care. In this way, the independent use of digital platforms as a primary source of information is not appropriate. To address this, the healthcare community must take active steps to produce and disseminate accurate, accessible, and engaging content.

## Funding

This work was supported by the Coordenação de Aperfeiçoamento de Pessoal de Nível Superior (CAPES, code 001).

## Conflicts of Interest

The authors declare no conflicts of interest.

## Ethics Statement

The author have nothing to report.

## Data Availability

The data that support the findings of this study are available on request from the corresponding author. The data are not publicly available due to privacy or ethical restrictions.
